# Admixed Phylogenetic Distribution of Drug Resistant *Mycobacterium tuberculosis* in Saudi Arabia

**DOI:** 10.1371/journal.pone.0055598

**Published:** 2013-02-01

**Authors:** Bright Varghese, Philip Supply, Caroline Allix-Béguec, Mohammed Shoukri, Ruba Al-Omari, Mais Herbawi, Sahal Al-Hajoj

**Affiliations:** 1 Department of Infection and Immunity, King Faisal Specialist Hospital and Research Centre, Riyadh, Saudi Arabia; 2 Genoscreen, Lille, France; 3 INSERM, U1019, Lille, France; 4 CNRS UMR 8204, Lille, France; 5 Institut Pasteur de Lille, Center for Infection and Immunity of Lille, Lille, France; 6 Univ Lille Nord de France, Lille, France; 7 National Biotechnology Centre, King Faisal Specialist Hospital and Research Centre, Riyadh, Saudi Arabia; University of Hyderabad, India

## Abstract

**Background:**

The phylogeographical structure of *Mycobacterium tuberculosis* is generally bimodal in low tuberculosis (TB) incidence countries, where genetic lineages of the isolates generally differ with little strain clustering between autochthonous and foreign-born TB patients. However, less is known on this structure in Saudi Arabia—the most important hub of human migration as it hosts a total population of expatriates and pilgrims from all over the world which is equal to that of its citizens.

**Methodology:**

We explored the mycobacterial phylogenetic structure and strain molecular clustering in Saudi Arabia by genotyping 322 drug-resistant clinical isolates collected over a 12-month period in a national drug surveillance survey, using 24 locus-based MIRU-VNTR typing and spoligotyping.

**Principal Findings:**

In contrast to the cosmopolitan population of the country, almost all the known phylogeographic lineages of *M. tuberculosis* complex (with noticeable exception of *Mycobacterium africanum*/West-African 1 and 2) were detected, with Delhi/CAS (21.1%), EAI (11.2%), Beijing (11.2%) and main branches of the Euro-American super-lineage such as Ghana (14.9%), Haarlem (10.6%) and Cameroon (7.8%) being represented. Statistically significant associations of strain lineages were observed with poly-drug resistance and multi drug resistance especially among previously treated cases (p value of < = 0.001 for both types of resistance), with relative over-representation of Beijing strains in the latter category. However, there was no significant difference among Saudi and non-Saudi TB patients regarding distribution of phylogenetic lineages (p = 0.311). Moreover, 59.5% (22/37) of the strain molecular clusters were shared between the Saudi born and immigrant TB patients.

**Conclusions:**

Specific distribution of *M. tuberculosis* phylogeographic lineages is not observed between the autochthonous and foreign-born populations. These observations might reflect both socially favored ongoing TB transmission between the two population groups, and historically deep-rooted, prolonged contacts and trade relations of the peninsula with other world regions. More vigorous surveillance and strict adherence to tuberculosis control policies are urgently needed in the country.

## Introduction

Tuberculosis (TB) still remains as a major, global public health problem. Worldwide, 10 million new cases of active TB and 1.8 million associated deaths are estimated to occur annually [1]. As judged by the information from available strain genotype databases [2], [3], [4], these impressive figures imply the existence and the circulation of at least tens of thousands strains of the causative agent *Mycobacterium tuberculosis* and its close relatives of the *M. tuberculosis* complex, such as *Mycobacterium bovis* and *Mycobacterium africanum*. Recent studies have shown an association between strain genetic lineages and TB patient geographic origin [2], [5], [6], [7], [8,] revealing a strong phylogeographical structuring of the pathogen. This association has been hypothesized to reflect the potential contribution of social factors limiting contacts between host population groups in some settings and/or potential adaptation of specific *M. tuberculosis* lineages to particular host populations, which have important implications, e.g. for new vaccine development [7], [9].

These associations have also been detected when studying large immigration urban centers in the Western world, such as San Francisco [7]; Brussels region [10]; and Montreal [8]; or Western countries such as Switzerland [11]. In such settings, *M. tuberculosis* strain genetic lineages are correlated with TB patient's country of birth or ethnic origin, the lineage distribution generally differs and little strain molecular clustering is generally found between native and foreign-born TB populations.

Here, we extended the investigation to another setting, Saudi Arabia, which is among the most dynamic immigration hubs on the globe. This country is a unique place, as it hosts approximately 8.4 million expatriates and annually receives another 10 million for Islamic rituals compared to a population of 18.7 million nationals. The majority of the visitors and expatriates come from high TB incidence countries, including but not limited to South- East Asia, Indian sub continent and Africa. Many of these foreign-born residents and pilgrims reside at least for one-week periods or much longer, in overcrowded and suboptimal living conditions, and in constant and repeated contact with citizens. These factors make the country a fertile land for TB transmission, potentially between different human groups. In accordance with this hypothesis, the high potential of upper respiratory diseases particularly TB was noticed during the Hajj pilgrimage [12], [13], [14], [15].

Therefore, we analyzed the *M. tuberculosis* strain genotype distribution among Saudi and non-Saudi TB patients from 8 different regions of the Kingdom of Saudi Arabia. This study took advantage of a recent nationwide drug-resistance survey, involving systematic genotyping of the drug resistant isolates. In contrast to a previous study for which spoligotyping alone was performed on isolates from the same regions collected in 2003–2005 [16], the present study also included the use of standard 24-locus MIRU-VNTR typing, known to provide superior discriminatory power for relevant analysis of epidemiological clustering and comparatively more accurate phylogenetic predictions [5], [17], [18].

## Results

### Study population

This study initially included 328 isolates that represent approximately 35% of the total estimate of drug resistant TB cases in the country during the study period. Six isolates were excluded from the study dataset based on the detection of double alleles in two or more MIRU-VNTR loci, indicating the simultaneous presence of two independent strains potentially due to mixed infection or contamination. The nationalities of the study subjects were composed of 51.9% Saudis and 48.1% non-Saudis. The non-Saudi population enrolled in the study comprised patients from 19 countries, mainly from Asia and Africa (Table 1). Of the total 214 (66.5%) cases were classified as “New” and 108 (33.5%) were “previously treated”, according to standard WHO guidelines [19], [20] (Table 2).

**Table 1:** Demographical summary of the study samples.

Parameters	Geographical Area								Total
	Dammam^1^	Riyadh^2^	Taif^3^	Jeddah^3^	Al-Baha^4^	Aseer^4^	Jizan^4^	Medina^3^	
Sample distribution	77(23.9)	122(37.9)	74(22.9)	25(7.7)	6(1.8)	12(3.7)	4(1.2)	2(0.6)	322
**Patient Origin**									
Saudi	30	73	37	12	3	7	3	2	167(51.9)
Non-Saudi	47	49	37	13	3	5	1	-	155(48.1)
South Asia^5^	25	19	11	5	-	1	-		61(18.9)
South East Asia^6^	12	21	7	4	1	1	-		46(14.3)
West Asia^7^	2	1	3	-	-	1	1		8(2.5)
Africa^8^	8	8	16	4	2	2	-		40(12.4)

1 Eastern Saudi Arabia.

2 Central Saudi Arabia.

3 Western Saudi Arabia.

4 Southern Saudi Arabia.

5 Bangladesh, India, Nepal, Pakistan, Sri Lanka,

6 China, Indonesia, Malaysia, Philippines.

7 Yemen.

8 Algeria, Chad, Egypt, Eritrea, Ethiopia, Nigeria, Senegal, Somalia, Sudan.

**Table 2:** Drug resistant pattern of the enrolled isolates.

Parameters	N (%)	Treatment history		
	N = 322	NEW^1^ (N/%)	Previously treated^2^ (N/%)	P value
**Drug resistance**		N = 214	N = 108	
Mono drug (N = 195)				<0.001
STR	90(27.9)	69(32.2)	21(19.4)	
INH	68(21.1)	44(20.6)	24(22.2)	
RIF	10(3.1)	7(3.3)	3(2.8)	
EMB	27(8.4)	22(10.3)	5(4.6)	
Polyresistance	54(16.8)	44(20.5)	10(9.3)	<0.001
MDRTB^3^	73(22.7)	28(13.1)	45(41.7)	<0.001
*(Pan resistance* 4 *)*	*41(56.2)*	*8*	*33*	

1 Newly diagnosed cases.

2 Patient received >1 month of anti TB drug therapy.

3 Multidrug resistant tuberculosis; resistant to at least isoniazid and rifampicin.

4 Pan-resistance was defined here as resistance to all four first-line drugs.

### Drug resistance patterns

Phenotypic drug susceptibility testing result showed that, out of the 322 retained isolates, 195 were mono-drug resistant, 54 poly-drug resistant and 73 multi-drug resistant (MDR)-TB (Table 2). Of the 73 MDR-TB isolates, 41 (56.2%) were pan-resistant, as defined here by resistance to all the tested first line drugs. Mono resistance to streptomycin (STR) was found to be highest (27.9%), followed by isoniazid (INH) (21.1%), ethambutol (EMB) (8.4%) and rifampicin (RIF) (3.1%) respectively. Mono drug resistance and poly drug resistance were found majorly (74.7%) among “new” cases (p<0.001). On the other hand MDRTB cases were found mainly (61.6%) among “previously treated” cases (p<0.001). We detected no association between patient's nationalities and isolate's drug resistance status (data not shown).

### Phylogenetic distribution

Analysis of the congruence between the MIRU-VNTR typing and the spoligotyping data for prediction of the phylogenetic lineages of the isolates was first performed by constructing a neighbor-joining tree based on MIRU-VNTR data followed by visual inspection of typical spoligotype signatures of the resulting strain groupings. The results revealed expected, consistent groupings, as seen from the fact that most isolates within the different groups shared typical spoligotype patterns and variants thereof (see e.g. the monophyletic grouping of the isolates with a typical Beijing spoligotype) (Figure S1).

In a second step, the MIRU-VNTR and spoligotyping data were submitted to MIRU-VNTRPlus, and subjected to a best-match, followed by a tree-based analysis, using the genotypes included in the database as references [19]. This analysis revealed the existence of 12 genetic lineages, which included almost all the known major branches of *M. tuberculosis* represented in MIRU-VNTRPlus database. Among these ones, Delhi/CAS (21.1%), EAI (11.2%) and Beijing (11.2%) lineages were the most prominent ones as well as the Ghana branch of the Euro-American super-lineage (14.9%) (Figure S1). Almost all other branches of this super-lineage were also detected, including Haarlem (10.6%), Cameroon (7.8%), and LAM (7.5%). Of note, we additionally found 15 (4.7%) cases of *M. bovis*, recognized as BCG Danish based notably on the identification of typical variant allele of locus 0580 (alias MIRU 04) [22], [23].

### Molecular cluster analysis

Twenty-four loci based MIRU-VNTR typing detected 207 distinct genotypes. Of these, 39 were clustered while 168 were unique. The clusters included each from 2 to 16 isolates, and a total of 155 isolates. In contrast, only 52 types were detected by spoligotyping, of which 36 were found in clusters including from 2 to 31 isolates and 16 were unique. As expected, only a minority of MIRU-VNTR-based clusters was subdivided by addition of secondary spoligotyping, resulting in a total of 221 types and reducing the number of clusters to 37 and the number of clustered isolates to 139 (Figure S2).

Analysis of correlation between strain clustering and drug resistance patterns showed a similar distribution of clustering among the mono (46.7%), and MDRTB isolates (50.7%), whereas poly-resistant (50%) isolates were slightly more clustered (Table 3). The strain clustering was comparatively slightly higher among Saudis (50.3%) compared to non-Saudis (45.8%) (Table 3).

**Table 3:** Molecular clusters found among the 322 study isolates.

Drug resistance	Total (N/%)	Clustered cases/proportion (N/%)^1^
Mono resistance		
STR	90(27.9)	47(52.2)
INH	68(21.1)	32(47.1)
RIF	10(3.1)	4(40)
EMB	27(8.4)	8(29.6)
Total	195(60.6)	91(46.7)
Poly resistance	54(16.8)	27(50.0)
MDRTB	73(22.7)	37(50.7)
Saudi	167 (51.9)	84(50.3)
Non Saudi	155(48.1)	71(45.8)

1 Molecular clustering identified based on MIRUVNTR and spoligo profiles.

**Figure 1:**
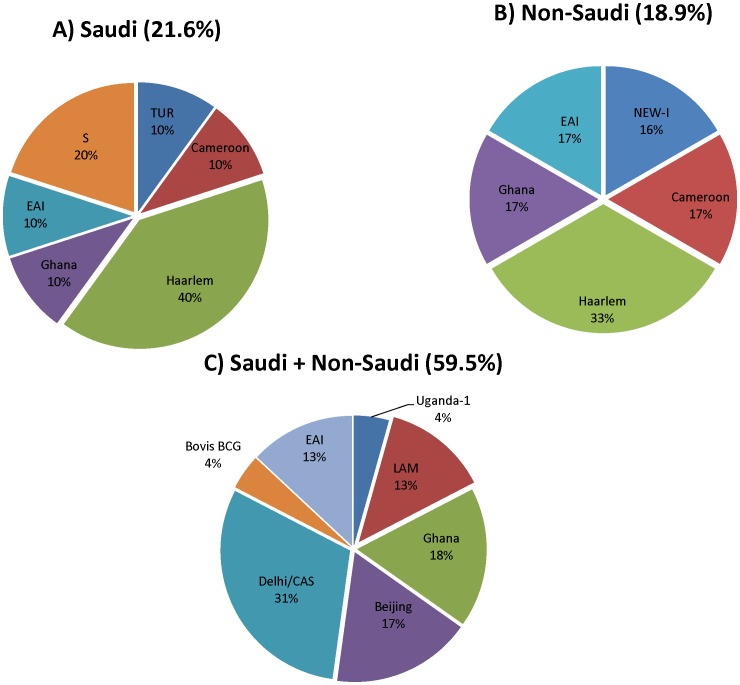
Distribution of strain clusters among the study groups. A) Saudi patients-only, B) non-Saudi patients-only and C) mixed strain molecular clusters. The distribution of strain clusters detected among the three categories is indicated above each diagram. Strain phylogenetic distribution with percentages of different lineages found within each cluster category is also shown. Strain molecular clusters were identified based on isolates sharing identical MIRU-VNTR types and spoligotypes.

We also analyzed the composition of the molecular clusters regarding the patient's nationalities. Interestingly, 59.5% (22/37) of the strain molecular clusters were composed both of Saudi and non-Saudi TB patients, while 18.9% of the clusters included only non-Saudi patients and 21.6% were composed exclusively of Saudi patients (Fig. 1).

### Lineages, molecular clustering, drug resistance and patient's nationality

We analyzed first the proportion of clustered isolates (as identified by the combination of MIRU-VNTR typing and spoligotyping data) in the different lineages identified. Interestingly, the highest proportion of clustered isolates was found for the Ghana lineage (68.7%), mostly due to the presence of the largest cluster overall (16 isolates) (Figure S2). The S lineage showed a clustering proportion of 75% but it only comprised a total of 8 isolates. Beijing (61.1%) and LAM (50.0%) also showed a relative over-representation of isolates in clusters compared to the average proportion (139/322; 43.2%), whereas Uganda-I (44.4%) and Haarlem (35.2%) were the two other lineages that showed relatively higher clustering rates close to the average.

All types of resistance patterns (mono resistant, poly-resistant and MDRTB) were found in all the above mentioned, mainly represented lineages, except for the *M. bovis* BCG cases where no MDR-isolates were detected as could perhaps be expected (Table 4). We analyzed the distribution of resistance patterns among the strain lineages after stratification per patient's history (i.e. new vs previously treated cases), to avoid a possible confounding factor. We found that poly-drug resistance and MDRTB were significantly associated with strain lineages, especially among the previously treated cases (p< = 0.001 for both types of resistance patterns, Table 4). Isolates of Beijing and EAI lineages appeared over-represented in the MDR category among the cases with a previous history of TB (Table 4).

**Table 4:** Association between drug-resistance and strain lineages.

Lineages	Total(n)	Drug Resistant Pattern			Drug Resistant Pattern		
		New (N = 214)			Previously Treated (N = 108)		
		Mono Drug^1^	Poly-Drug	MDRTB	Mono Drug	Poly-Drug	MDRTB
Delhi/CAS	68	29(13.5)	3(1.4)	3(1.4)	6(5.6)	18(16.7)	9(8.3)
Ghana	48	34(15.9)	2(0.9)	-	-	-	12(11.1)
EAI	36	19(8.9)	3(1.4)	-	-	-	14(12.9)
Beijing	36	16(7.5)	-	4(1.9)	-	5(4.6)	11(10.2)
Haarlem	34	21(9.8)	8(3.7)	-	1(0.9)	1(0.9)	3(2.8)
Cameroon	25	13(6.1)	-	2(0.9)	4(3.7)	1(0.9)	5(4.6)
LAM	24	15(7.0)	2(0.9)	-	-	4(3.7)	3(2.8)
Bovis BCG	15	13(6.1)	2(0.9)	-			-
Others^2^	36	23(10.7)	2(0.9)	-	1(0.9)	3(2.8)	7(6.5)
*P value*		0.438	0.046	0.004	0.006	0.001	0.000

1 Mono drug resistance was used as the closest reference to a pan-sensitive status.

2 Uganda-I (N = 9), S (8), X (7), New I (n = 5),), TUR (n = 5), Unknown (n = 2).

**Figure 2:**
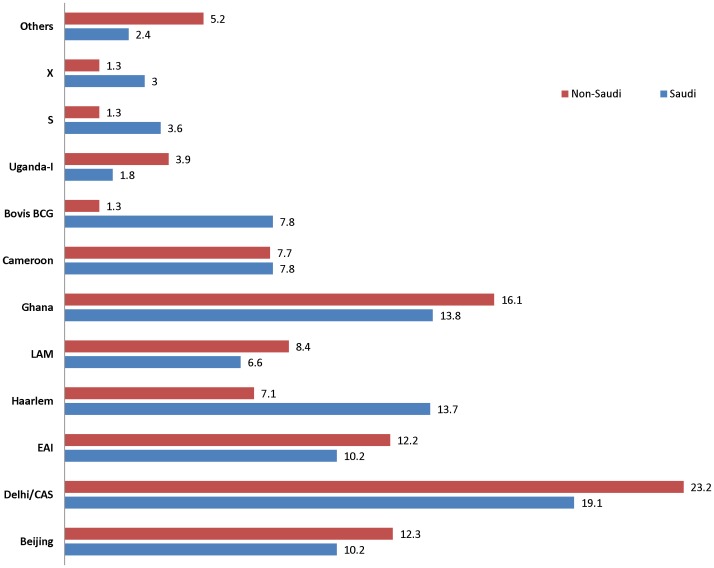
Distributions of *M. tuberculosis* complex strain lineages among Saudi and non-Saudi cases. Lineages identified as described in text were segregated as per “Saudi” and “Non-Saudi” nationalities. Bar diagrams show the proportion (in percentage) of each lineage in each of these two groups. The lineages TUR, NEW-I and URAL were clubbed under the label “Others” as the corresponding isolates are very few in number.

In contrast, analysis of the distribution of phylogenetic lineages among Saudi and non-Saudi patients showed no statistical association (p = 0.311) of patient's nationality (Saudi vs non-Saudi) with lineage (Fig. 2). Proportions of each lineage in the two patient groups were similar, with the expectable exception of *M. bovis* corresponding to the Danish BCG vaccine strain restricted mainly to Saudi children. Along the same lines, mixed molecular clusters (ie. composed of both Saudi and non-Saudi patients) were seen among most of the strain lineages except NEW-I, Haarlem, Cameroon, EAI, and TUR (Fig. 1). The relative proportions of lineages found among the different provinces were also overall similar, except that EAI and Cameroon tended to be more represented in Taif and in Dammam, respectively (Figure S3).

## Discussion

Two main findings were obtained from the present study. Firstly, a particular broad distribution of *M. tuberculosis* strain lineages was observed among the 322 isolates collected from 8 regions of Saudi Arabia. This is completely in line with intense human pilgrimage and cosmopolitan migration into the country. Secondly and less expectedly, we observed very similar strain lineage distributions among Saudi and non-Saudi TB patients and a majority of strain molecular clusters were shared by patients of both groups, in striking contrast to large immigration centers of the Western hemisphere where native and foreign-born TB patients show more specific, respective strain populations.

Genotyping allowed us to detect in this strain collection almost all the major branches of *M. tuberculosis* represented in MIRU-VNTRPlus database. This wide spectrum of the pathogen's population clearly reflects the diversity of geographic origins in the human host population in the Kingdom. Saudi Arabia hosts a huge population of migrants from worldwide, and receives 10 million visitors for Islamic rituals every year. Both categories of population are coming in majority from high TB endemic countries, and accordingly have an impact on TB transmission [13], [24], [25]. Migrants constitute a major proportion of the TB cases annually reported in the country according to a recent study [26].

The detected predominance of Delhi/CAS (alias East-African Indian by Large Sequence Polymorphism nomenclature), Beijing (alias East-Asian) and EAI (alias Indo-Oceanic) lineages, which together formed 43.5% (N = 140) of the total studied cases, is consistent with the fact that majorities of the expatriates are coming from the Indian subcontinent (India, Pakistan, Sri-Lanka, Bangladesh, and Nepal) and South East Asia (Indonesia, Philippines and Malaysia), where these lineages prevail [27], [28], [29], [30], [31], [32]. Along the same lines, the identification of most branches of the Euro-American super-lineage is also coherent with the presence of large numbers of expatriates/visitors from different parts of Africa and Europe, where these branches are widespread [6]. Furthermore, the identification of 15 *M. bovis* BCG strains with resistance mainly to INH (13 cases), INH and STR (2 cases) is also fully in concordance with a recent report which showed an outbreak of lymphadenitis among Saudi children who were vaccinated with the Danish SSI 1331 strain [33]. The INH resistance of the BCG SSI strain was also observed previously [34].

One branch of the Euro-American lineage, Ghana, was particularly well represented (48 isolates; 14.9%) and showed both the largest molecular cluster (16 isolates) and the highest proportion of clustered isolates (33/48; 68.7%) among all lineages identified. It is worth mentioning that, for this branch, a majority of the isolates (29/48, 60.4%) were obtained from non-Saudi patients. Although the detected molecular clusters might not all reflect in-country TB transmission but could also reflect importation of endemic strains from the country of origin, particularly high proportion of clustering for Ghana isolates might mirror crowded living, lower average socio economical status, non adherence to timely TB notification and treatment among the concerned residents, favoring enhanced TB transmission within this particular community.

The finding that isolates of the Beijing lineage tended to be over-represented among MDR-TB cases compared to mono-drug resistant cases especially among previously treated patients in Saudi Arabia is also in keeping with other reports showing frequent association of this lineage with MDR-TB in different parts of the world [35]. EAI also appeared over-represented in this category. Association of both EAI and Beijing lineages with MDR has been noticed in some countries from which many expatriates in Saudi Arabia originate, such as Pakistan [36].

While the detection of a wide spectrum of pathogen's genetic lineages was predictable given the cosmopolitan influence on the country, it was less expected to find a similar strain lineage distributions among Saudi and non-Saudi TB patients (Figure 2) and a majority of strain molecular clusters (22 out of 37; 59.5%) shared by patients of both groups. These findings differ from those reported for large immigration centers of the Western hemisphere [6], [7], [8], [10], [11]. In these settings, the Euro-American lineage strongly predominates among the isolates from in-country-born patients of the local ethnic groups, and among these patients, strains from other lineages strongly associated to foreign-born patients from other continents are rarely found. Moreover, in these studies, mixed molecular clusters (i.e. including isolates from both native and foreign-born patients) constituted only a minority of the total number of patient strain clusters. For example, only 3 out of 53 strain clusters identified by 24-locus MIRU-VNTR typing and spoligotyping in a population-based study in Brussels comprised Belgian-born and foreign-born settled and unsettled immigrant patients [10]. In a Swiss study, the percentage of such mixed clusters (identified by using the same genotyping markers) was 31% [11].

The present study has some limitations compared to the studies mentioned above. In contrast to the latter ones, it included only drug-resistant isolates, linked to the opportunity to collect such isolates from most parts of the country in the frame of a recent nationwide drug-resistance survey. However, it is unlikely that this design has created a bias leading to the observed differences in the strain genetic lineage and cluster distributions among native and foreign-born TB patient populations. The previous studies did not notice a divergent trend regarding these distributions that would be specific to drug-resistant isolates compared to drug-sensitive isolates. Also, the present study included a convenience-based sampling linked to a national survey over a 12-month period, while the previous studies were either also based on sampling or were population-based at a regional or national level, on longer time periods (from 3 to 11 years). Again, we do not believe that these sampling differences could have generated a bias that could account for the observed differences in strain cluster and phylogenetic distributions. Although not exhaustive, our strain collection represents a substantial proportion (approximately 35%) of the total estimate of drug resistant TB cases from 8 out the 9 regional TB reference laboratories in the country over the study period. With respect to differences of study time periods, no changes in mixed clustering parameters or in association between strain lineages and host populations were noticed over time in the previous studies; associations between specific host and pathogen populations appeared stable [7]. Lastly, the HIV status of the TB patients involved in this study was not available. It has been suggested that, although pathogen's lineages could be less adapted to transmit and cause disease in immune-competent members of allopatric human populations, they could do so in the context of impaired host immune system [6]. However, the very low estimated prevalence of HIV infection in the general Saudi population (21/100,000 in 2010) [37] argues against a significant role of this factor in our study.

Instead, we believe that the admixed phylogenetic and cluster distributions specifically observed in this study might reflect a heavy weight of demographical, sociological and geo-historical factors proper to Saudi Arabia. Firstly, taking into account the stable yearly influx of pilgrims in addition to the resident migrants, the almost one-to-one proportion of foreign-born vs locally born people in the country is massively higher than in the Western settings indicated above. Secondly, modalities of social mixing among the two population groups probably differ between the respective settings. While socio-economical and cultural barriers can reduce contacts between foreigners and in-country-born populations in the previously studied settings, a large part of the important expatriate population in the Kingdom occupy household functions in everyday contact with Saudi families. Both specificities (demography and social mixing) plausibly augment opportunities for TB transmission between Saudis and non-Saudis. A third, important specific trait of the Arabian peninsula is its central geographical position at the cross-roads between Asia and Africa, and it's very old and intense trade relations with these two world regions, dating back at least to 1000 A.D [38], [39]. Thus, the broad phylogenetic distribution of *M. tuberculosis* strains in the Saudi population could be linked not only to the influence of present/recent contacts but as well to historically deeper-rooted, permanent interactions with the African and Asian human populations.

Importantly, the observed admixture of *M. tuberculosis* phylogeographic lineages and strains between Saudi and non-Saudi TB patients does not disqualify the hypothesis of adaptation of specific *M. tuberculosis* lineages to particular host populations. If such adaptation exists, we hypothesize that the specificities described above having favored intense, long-lasting interactions between Saudi and non-Saudi populations could have significantly eroded specific associations that are seen elsewhere between host and pathogen populations.

More practically, this substantial admixture, also seen at the strain cluster level and suggestive thus of substantial ongoing TB transmission between population groups, calls for a more vigorous surveillance and adherence to TB control policies in the country.

## Materials and Methods

### Study population

Saudi Arabian national TB control program is well supported by nine regional TB reference laboratories which stand as the major TB diagnostic facilities for the whole country. All the new cases from the clinics and hospitals are reported to these central facilities in each province. The study samples were collected from 8 of 9 regional TB laboratories during a twelve-month period, from July 2009 to June 2010. Only *M.tuberculosis* isolates initially reported as drug resistant by the regional laboratories were enrolled. Although representing a substantial proportion of the estimated drug-resistant TB cases over the study period (see Results), the sample collection was mostly based on convenience, ie; the study sites only sent drug resistant isolates growing well in culture. The processing of the study isolates was conducted at different time intervals and by different technical staffs. To avoid cross-contamination during isolation and culturing, all the manipulations were done individually in biological safety cabinets equipped with a negative-pressure system.

### Drug susceptibility testing

The drug susceptibility to the first line drugs STR, INH, RIF and EMB was carried out by using the BACTEC MGIT 960 culture system (Becton Dickinson, CA, USA). Mono, poly and multiple-drug resistant isolates were defined as isolates resistant to a single, at least two (other than both INH and RIF), and to at least both INH and RIF drugs, respectively, according to standard definitions.

### Molecular typing

The genomic DNA was extracted using QIAamp DNA Mini Spin column [QIAGEN, Hilden, Germany] method according to the manufacturer's instructions. Molecular fingerprinting of the study isolates were carried out by using the standardized 24 loci based MIRU-VNTR typing (Genoscreen, Lille, France) and Spoligotyping (Ocimum Biosolutions, Hyderabad, India) according to internationally standardized protocols [18], [40]. Extraction of genomic DNA was performed in batches. PCR was carried out in an independent PCR workstation dedicated to this work, and UV and manual decontamination were performed after each step. Negative controls were systematically included with PCR steps of MIRU-VNTR typing and spoligotyping. Six isolates displayed double alleles in two or more loci suggestive of mixed genotypes, which could reflect either mixed infection or contamination. These six samples were excluded from the analysis.

### Data analysis

The drug susceptibility results were defined into three major groups, an isolate resistant to single drug (mono drug resistance), more than one drug (poly resistance) and resistance to at least both INH and RIF (MDRTB). A patient who received anti-tuberculosis therapy for >1 month was considered as “previously treated”, and for <1month as “New”. The spoligotyping results were converted into numerical octal codes. The alleles of MIRU-VNTR types were initially determined by using Genemapper V-4.0 (Applied Biosystems, CA, USA) and data were compiled by the MIRU-VNTR Data manager (Genoscreen, Lille, France). MIRU-VNTR and spoligotyping data were submitted to the international online database (www.miru-vntrplus.org) to assign the lineages according to the previously described strategy, combining best-match- and tree-based analysis [3], [21]. Molecular clustering of the isolates was determined by constructing a dendogram based on the combination of MIRU-VNTR and spoligotyping data, using the unweighted pair group method using average linkages and the categorical coefficient. A strain cluster was defined as two or more isolates sharing completely identical genotypes. The statistical analysis of the results was carried out by using the software package SPSS V-19.0 (IBM, USA). Associations with mono, poly or multidrug resistance were calculated by chi square analysis. The *p* value<0.05 was considered as statistically significant.

## Supporting Information

Figure S1 **Genetic lineages among 322 drug resistant** ***M.tuberculosis*** **isolates from Saudi Arabia.** The UPGMA tree was built based on MIRU-VNTR typing data. The spoligotypes data are shown in correspondence to the MIRU-VNTR-based groupings for visualization of the concordance with spoligotypes signatures, or variants thereof, typical of different genetic lineages. Lineages were assigned by best-match followed by tree-based analysis under MIRU-VNTRPlus database. Lineage information followed by isolate identifier number is boxed and colored according to each lineage.
(TIF)

Figure S2 **Molecular clusters of** ***M. tuberculosis*** **complex isolates based on MIRUVNTR and Spoligotypes.** The UPGMA tree was built and clusters were identified based on isolates sharing identical MIRU-VNTR types and spoligotypes.
(TIF)

Figure S3 **Strain lineage distribution in the major provinces.** The major lineages distributed through the main provinces/study sites are illustrated. The areas of Jizan, Al-Baha and Medina were clubbed as “Others” as the respective isolate numbers were very low. The lineages TUR, NEW-I, Uganda-I, S, X, URAL are also labeled together as “Others” for the same reasons.
(TIF)
