# Supplementary figures and images for: Admixed Phylogenetic Distribution of Drug Resistant Mycobacterium tuberculosis in Saudi Arabia
Source: PLoS One. 2013 Feb 1;8(2):e55598. doi: 10.1371/journal.pone.0055598 (PMC3562184; doi:10.1371/journal.pone.0055598)

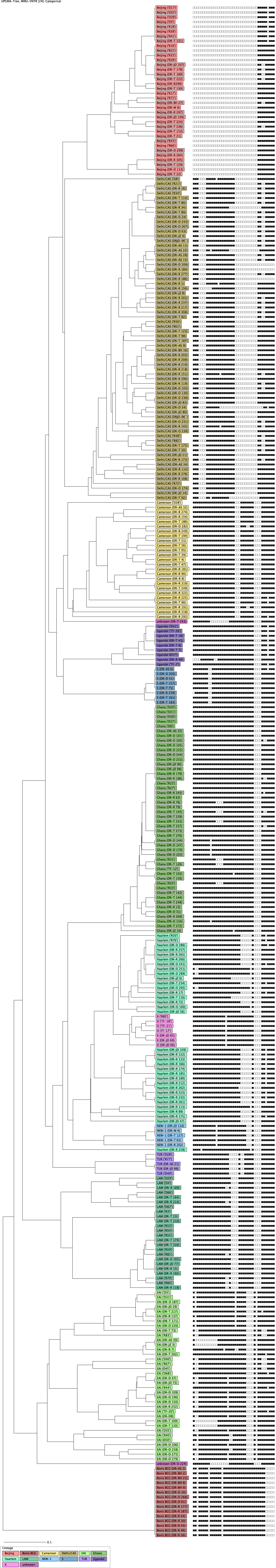

Supplement: Figure S1 — Genetic lineages among 322 drug resistant M.tuberculosis isolates from Saudi Arabia. The UPGMA tree was built based on MIRU-VNTR typing data. The spoligotypes data are shown in correspondence to the MIRU-VNTR-based groupings for visualization of the concordance with spoligotypes signatures, or variants thereof, typical of different genetic lineages. Lineages were assigned by best-match followed by tree-based analysis under MIRU-VNTRPlus database. Lineage information followed by isolate identifier number is boxed and colored according to each lineage. (TIF) [file pone.0055598.s001.tif]

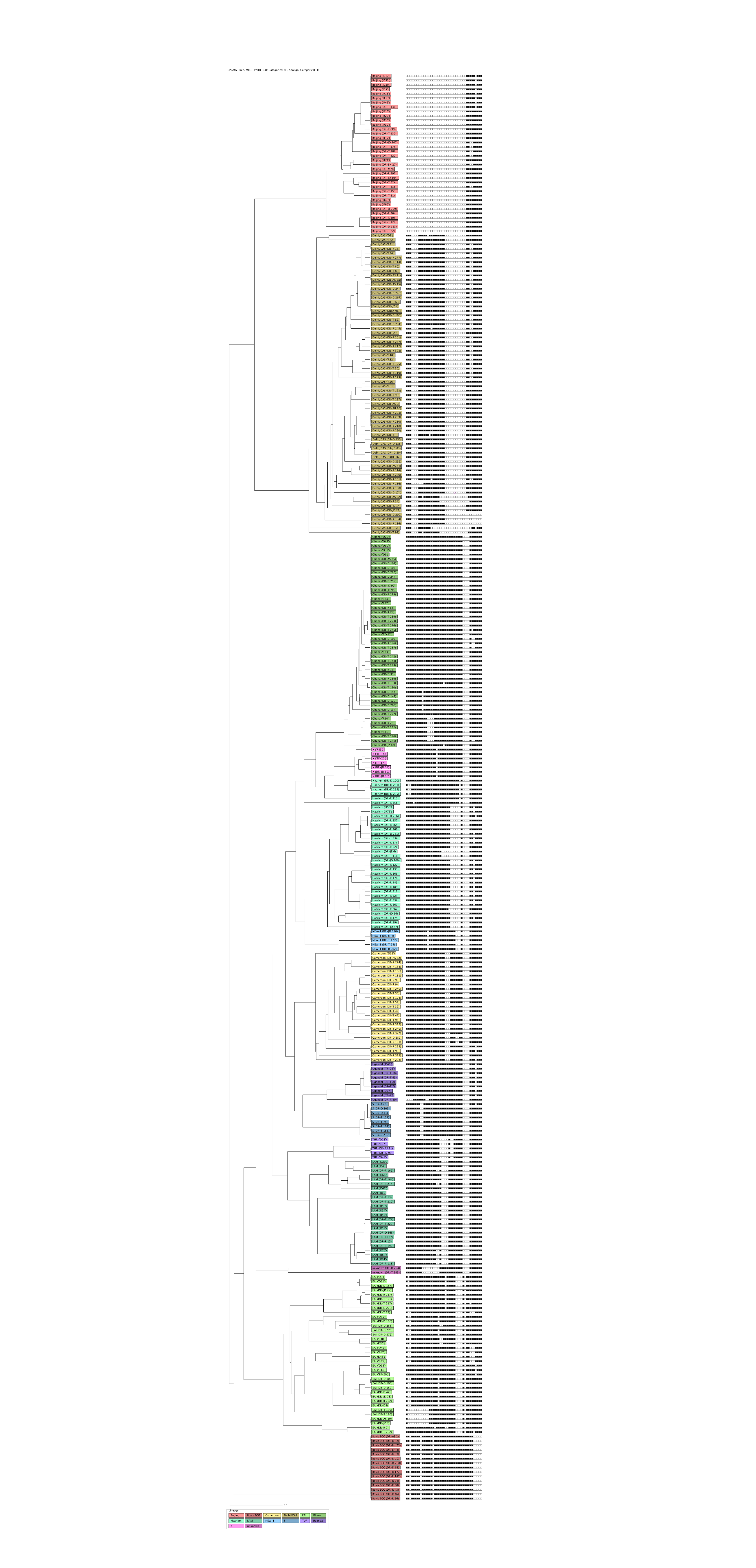

Supplement: Figure S2 — Molecular clusters of M. tuberculosis complex isolates based on MIRUVNTR and Spoligotypes. The UPGMA tree was built and clusters were identified based on isolates sharing identical MIRU-VNTR types and spoligotypes. (TIF) [file pone.0055598.s002.tif]

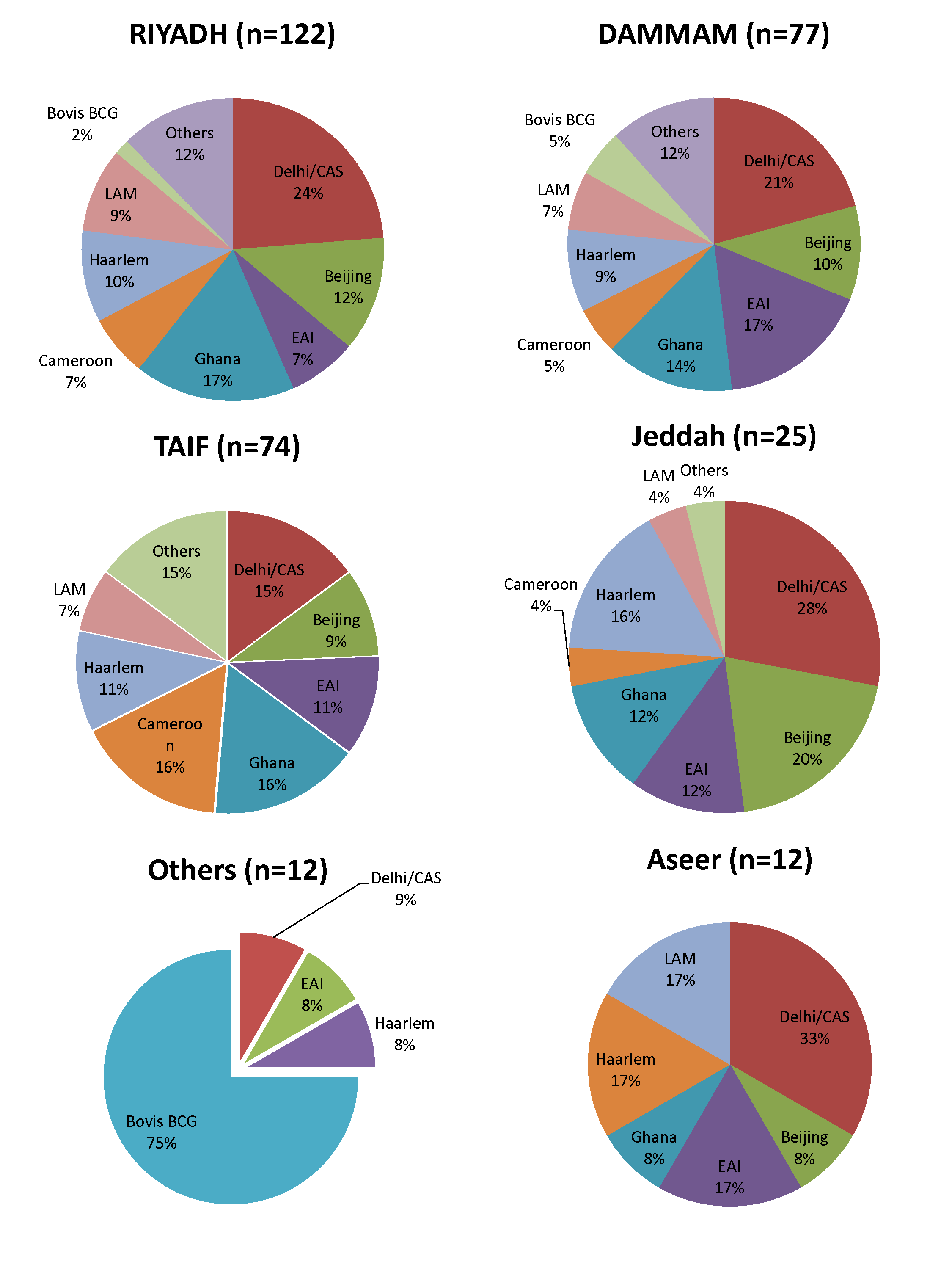

Supplement: Figure S3 — Strain lineage distribution in the major provinces. The major lineages distributed through the main provinces/study sites are illustrated. The areas of Jizan, Al-Baha and Medina were clubbed as “Others” as the respective isolate numbers were very low. The lineages TUR, NEW-I, Uganda-I, S, X, URAL are also labeled together as “Others” for the same reasons. (TIF) [file pone.0055598.s003.tif]
